# The ticking clock: does actively making an enhanced care team aware of the passage of time improve pre-hospital scene time following traumatic incidents?

**DOI:** 10.1186/s13049-020-00726-9

**Published:** 2020-04-29

**Authors:** L. Curtis, E. ter Avest, J. Griggs, J. Wiliams, R. M. Lyon

**Affiliations:** 1 Air Ambulance Kent, Surrey and Sussex, Redhill Aerodrome, Redhill Airfield, Redhill, Surrey, RH1 5YP UK; 2 Department of Emergency Medicine, University Hospital Groningen, Groningen, the Netherlands; 3 School of Health Sciences, University of Surrey, Guildford, UK; 4 South East Coast Ambulance Service NHS Foundation Trust, Crawley, UK

**Keywords:** Trauma, HEMS, Scene time, Prompting timer

## Abstract

**Introduction:**

Pre-hospital enhanced care teams like Helicopter Emergency Medical Services (HEMS) are often dispatched to major trauma patients, including patients with traumatic brain injuries and those with major haemorrhage. For these patients, minimizing the time to definitive care is vital. The aim of this study was to determine whether increased awareness of elapsed on scene time produces a relevant time performance improvement for major trauma patients attended by HEMS, and weather introducing such a timer was feasible and acceptable to clinicians.

**Methods:**

We performed a prospective cohort study of all single casualty traumatic incidents attended by Air Ambulance Kent Surrey Sussex (AAKSS) between 15 October 2016 and 23 May 2017 to test if introduction of a prompting scene timer within the service resulted in a reduction in pre-hospital scene times.

**Results:**

The majority of the patients attended were male (74%) and sustained blunt trauma (92%). Overall, median scene time was 25.5 [IQR16.3] minutes before introduction of the scene timer and 23.0 [11.0] minutes after introduction, *p* = 0.13). Scene times for patients with a GCS < 8 and for patients requiring prehospital anaesthesia were significantly lower after introduction of the timer (28 [IQR 14] vs 25 [1], *p* = 0.017 and 34 [IQR 13] vs 28 [IQR11] minutes, *p* = 0.007 respectively). The majority of clinicians felt the timer made them more aware of passing time (91%) but that this had not made a difference to scene time (62%) or their practice (57%).

**Conclusion:**

Audible scene timers may have the potential to reduce pre-hospital scene time for certain single casualty trauma patients treated by a HEMS team, particularly for those patients needing pre-hospital anaesthesia. Regular use of on-scene timers may improve outcomes by reducing time to definitive care for certain subgroups of trauma patients.

## Background

In many western countries, trauma is the leading cause of death in those under 40 years of age [2]. For several decades one of the corner stone principals of pre-hospital trauma care has been to improve survival by reducing the time taken to deliver patients from the point of injury to definitive care (“scoop and run”). Studies attempting to explore and establish the relationship between pre-hospital time and patient outcome (mortality) in the civilian setting have shown largely equivocal results [3, 4]. However, a clear mortality reduction associated with shorter scene times has been demonstrated for more discreet trauma cohorts, such as patients suffering from penetrating trauma [5–7] and traumatic brain injury (TBI) [8, 9].

Enhanced pre-hospital care teams, such as Helicopter Emergency Medical Services (HEMS), are often dispatched to major trauma patients, including patients with TBI and patients with penetrating injuries. Enhanced care teams have the ability to perform critical care interventions at the scene (“stay and play”) beyond the capability of other pre-hospital responders. However, performing enhanced pre-hospital interventions can increase scene time, especially when advanced interventions, such as rapid sequence induction (RSI) have to be performed [1, 10–12]. Close monitoring of scene times is therefore advisable, especially as clinicians generally tend to underestimate the time elapsed whilst performing complex clinical interventions [13, 14].

Previous studies have shown that temporal awareness improves performance [15] and reduces the time taken to deliver clinical interventions [16]. One way to improve temporal awareness, is through the use of a prompting timer. The aim of this study was to determine whether the introduction of an audible pre-hospital scene timer, which alerted clinicians to elapsed time on scene, produced a relevant time performance improvement, and weather introducing such a timer was feasible and acceptable to clinicians.

## Methods

### Study design

We performed a prospective cohort study of all single casualty traumatic incidents attended by Air Ambulance Kent, Surrey Sussex (AAKSS) between 15 October 2016 and 23 May 2017, to test if making a HEMS team actively aware of the passage of time using a prompting scene timer does improve the prehospital scene time of traumatic incidents.

To test the hypothesis that making a HEMS team actively aware of the passage of time does improve the pre-hospital times of traumatic incidents, we performed a pre- and post change evaluation of the introduction of a prompted scene timer. Based on previous publications [15] and consensus opinion from within the service, an observed effect size in excess of 15% was felt to be indicative of significant change. In order to detect this with a power of 80%, and a mean pre-intervention scene time of 25 ± 11 min our sample size was set at > 125 patients in each group. Subgroup analysis was pre-specified for potentially confounding factors influencing scene time (age, mechanism of injury, GCS, interventions on scene, mode of transport HEMS and time of day).

### Setting

AAKSS is a Helicopter Emergency Medical Service (HEMS) covering three counties (9000 km^2^) in the southeast of the UK with a resident population of approximately 4.6 million people. This service operates 24 h a day and its clinical team consists of a doctor and paramedic. Paramedics have several years of critical care experience, and doctors are in their last stage of specialty training or consultant level. Both doctors and paramedics follow an intensive prehospital induction course, and have a supervised sign-off period before they start working independently. HEMS is deployed to patients suffering suspected critical, or life threating injury or illness. Annually, this service responds to in excess of 2500 incidents.

### Intervention

After an initial period wherein scene times were measured without feedback to the crews, a prompting timer was introduced. The timer system involved was a GYMBOSS interval timer being placed on the services’ dispatch desk. The timer was started by the dispatcher, once notified by the HEMS team that they had arrived on scene. After every 5 min the GYMBOSS sounded an alarm prompting the dispatcher to make a radio call to the HEMS team stating the time elapsed. The process continued until the team departed the scene with the patient.

### Study population

Incidents were selected for inclusion using a consecutive sampling strategy of all single casualty incidents involving traumatic injury attended by the service during the project period. Incidents where patients required extrication were excluded, as the process was beyond the direct control of the HEMS team and inclusion would potentially confound the true effect of the time. Incidents where the HEMS team did not convey the patient from scene to hospital, were also excluded, as it was not feasible to establish scene times for these patients. Finally, a scene time of less than 5 min was an exclusion criterium, as scene times below 5 min could not be affected by a 5 min interval timer.

### Outcome measures

The primary outcome was defined as the time spend on scene, from arrival on the patients’ side until leaving scene.

The secondary outcome was the feasibility and acceptability of using the prompting scene timer as expressed on a 5-point scale.

### Data acquisition

For both pre- and post-implementation periods timings were collected and entered real-time in the electronic patient clinical record system (HEMSbase 2.0, Medic One Systems Ltd., UK) by the HEMS dispatcher in the ambulance control centre. Information about potential confounders affecting scene time was collected for both periods from HEMSbase. The following data were retrieved: Age, mechanism of injury [road traffic collision/ other], patient GCS, interventions on scene [prehospital anesthesia/thoracostomy/thoracotomy/blood product administration], mode of transport [car/helicopter] and time of day [day/night].

At the conclusion of the post change period, all HEMS-team members were contacted and invited to complete an anonymous electronic 10-question structured questionnaire to determine the effectiveness, perception and user acceptability of the scene timers (Supplementary file 1).

### Ethical considerations

This project met the National Research Ethics Service (NRES) definition of service evaluation audit (NRES, 2009) and therefore did not require ethical approval. Ethics approval however was sought and was granted for the conduct of the staff survey by the University of Hertfordshire Research Ethics Committee.

### Statistical analysis

Shapiro Wilk tests were performed to assess normal distribution and residual plots were drawn to assess linearity of data. Descriptive statistics are given as numbers [%] or median [IQR]. Comparisons across groups for baseline characteristics were made using Fisher’s exact test and Mann-Whitney U test where appropriate. Scene-time comparisons were made by Mann-Whitney U test or independent t-test where appropriate. Per protocol scene time comparisons were made using univariate analysis of variance (GLM) with mode of transportation (helicopter/ambulance) as a co-factor, with pre-specified subgroup analysis for potentially confounding factors. Survey data was summarised using descriptive statistics. Missing values are reported in the results section of the manuscript according to the STROBE guideline [17]. A *p*-value < 0.05 was regarded as statistically significant. All statistical analyses were conducted using IBM SPSS 23.0 for Windows statistical package.

## Results

### Study population

**Fig. 1:**
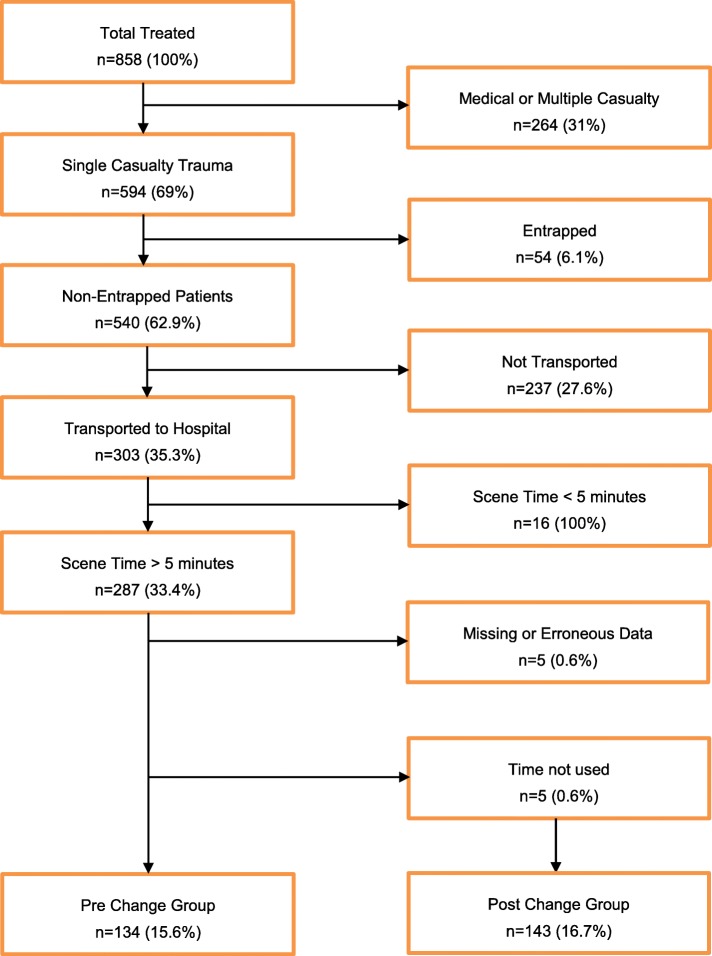
Inclusion Criteria

During the study period, a total of 858 patients were attended, of which 594 (69%) met the inclusion criteria. 54 patients (9%) who required extrication were excluded, as were 237 (40%) patients who were not conveyed from scene by the HEMS team. 16 patients (3%) had a scene time less than 5 min, and for 5 patients (1%) scene times were either missing or erroneous (Fig. 1). Scene time data were captured for a total of 282 patients: 134 patients in the period before introduction of the scene timer, and 148 in the period after introduction. For 5 patients in the post-introduction group the protocol was violated and the timer had not been started. These were excluded from the further (per protocol) analysis.

Table 1 shows the study population characteristics. Before- and after introduction of the scene timer, the majority of patients was male, involved in RTC’s- or accidental injury resulting, and sustained resulting blunt trauma. Frequency distribution of mechanism of injury, type of injury, moment of injury, and interventions performed on scene was equal for both study periods. In the period after introduction of the scene timer however, significantly more patients were transported to hospital by helicopter compared to by ground ambulance (37% vs 59%, *p* < 0.001). As overall scene time for patients transported to hospital by helicopter was longer than for those being transported by road (25 min [IQR 12.3] vs 22 min [15.2], *p* < 0.001), mode of transport was identified as a relevant co-variate for subsequent analysis of variance of scene time.

**Table 1:** Characteristics of the study population

	No Scene Timer		Scene Timer		*p*
	n	%	n	%	
**Gender**					
Male	101	75.4	104	72.7	0.68
Female	33	24.6	39	27.3	
**Age**					
Median [IQR]	49 [42]		50 [41]		0.53
**Adult/ Child**					
Age > =18	122	91.0	130	90.9	0.99
Age < 18	12	9.0	13	9.1	
**Mechanism of Injury**					
Accidental Injury	53	39.6	48	33.6	0.59
Assault	13	9.7	11	7.7	
Exposure	2	1.5	2	1.4	
Intentional Self-Harm	5	3.7	12	8.4	
Other Transport	1	0.7	0	0.0	
RTC	53	39.6	62	43.4	
Sport/Leisure	7	5.2	8	5.6	
**Trauma Type**					
Blunt Trauma	123	91.8	132	92.3	0.99
Penetrating Trauma	11	8.2	11	7.7	
**Time of Day**					
Day	105	78.4	103	72.0	0.27
Night	29	21.6	40	28.0	
**GCS**					
median [IQR]	13 [8]		14 [8]		0.32
GCS > 8	89	66.4	104	72.7	0.30
GCS < =8	45	33.6	39	27.3	
**RSI**					
RSI	44	32.8	42	29.4	0.60
No RSI	90	67.2	101	70.6	
**Transfusion**					
Blood products	15	11.2	15	10.5	0.99
No Blood products	119	88.8	128	89.5	
**Thoracostomy**					
Thoracostomy	11	8.2	16	11.2	0.43
No Thoracostomy	123	91.8	127	88.8	
**CPR**					
CPR	9	6.7	8	5.6	0.84
No CPR	125	93.3	135	94.4	
**Transport to Hospital**					
Helicopter	49	36.6	84	58.7	0.001
ambulance	85	63.4	59	41.3	

*GCS* Glasgow Coma Scale, *RSI* Rapid sequence induction, *CPR* cardiopulmonary resuscitation

### Effect of prompted timer

Univariate analysis of variance was performed with mode of transportation as a co-factor to investigate the effect of the prompted timer on scene times. Overall median scene times were slightly lower after implementation of the prompted timer (23.0 [IQR 11.0] minutes vs 25.5 [IQR 16.3]). However, the difference did not meet the pre-specified clinically relevant difference of 15%, and statistical significance was not reached (*p* = 0.128), Table 2.

**Table 2:** Effect of a prompting scene timer on scene times for single casualty traumatic incidents attended by HEMS

Group	No Scene Timer			Scene Timer			Difference in mins.	% Change	Significance *p*
	Number	Median (Mean)	IQR (SD)	Number	Median (Mean)	IQR (SD)			
All Incidents	134	25.5	16.3	143	23.0	11.0	2.5	9.8	0.13
Adults (age > =18)	122	26.0	16.3	130	23.0	11.3	3.0	11.5	0.20
Children (age < 18)	12	(24.4)	(12.1)	13	(22.5)	(7.7)	1.9	7.7	0.45
RTC	53	27.0	17.0	62	23.0	13.5	4.0	14.8	0.13
Non-RTC Mechanism	81	25.0	18.5	81	24.0	11.0	1.0	4.0	0.27
Blunt Trauma	123	27.0	17.0	132	24.0	10.8	3.0	11.1	0.08
Penetrating Trauma	11	8.0	5.0	11	11.0	14.0	−3.0	−37.5	0.64
Day	105	27.0	16.0	103	23.0	12.0	4.0	14.8	0.08
Night	29	(24.1)	(13.0)	40	(24.9)	(9.1)	−0.9	−3.6	0.89
GCS > 8	89	22.0	18.0	104	23.0	12.8	−1.0	− 4.5	0.92
GCS < =8	45	(29.7)	(10.1)	39	(25.2)	(8.1)	4.5	15.1	0.028
RSI	44	34.0	13. 0	42	28.0	11.0	6.0	17.6	0.007
No RSI	90	19.0	14.3	101	21.0	11.0	−2.0	−10.5	0.96
Blood products	15	(27.3)	(12.1)	15	(30.8)	(8.8)	−3.5	−12.9	0.37
No Blood products	119	25.0	16.0	128	23.0	11.0	2.0	8.0	0.21
Thoracostomy	11	(32.5)	(15.7)	16	(31.9)	(12.5)	0.5	1.6	0.93
No Thoracostomy	123	25.0	17.0	127	23.0	10.0	2.0	8.0	0.25
CPR	9	(26.7)	(9.9)	8	(29.1)	(13.3)	−2.5	−9.2	0.76
Helicopter Transport	49	27.0	13.5	84	24.0	11.0	3.0	11.1	0.28
Ambulance Transport	85	23.0	19.0	59	21.0	11.0	2.0	8.7	0.50

*RTC* road traffic collision, *GCS* Glasgow coma scale, *RSI* rapid sequence induction, *CPR*cardiopulmonary resuscitation;

Subgroup analysis demonstrated a significant difference in scene time for patients who underwent prehospital anaesthesia and for patients with a low GCS. For these teams attending these patients, the prompted timer was associated with a reduction in scene time of respectively 6 min (34 [IQR 13] vs 28 [IQR11] minutes, *p* = 0.007) and 3 min (28 [IQR 14] vs 25 [10] minutes, *p* = 0.017), Table 2.

### Feasibility and acceptability

Of the 45 clinicians who took part in the project (doctors *n* = 25, paramedics *n* = 20) anonymised survey responses were received from 21 (doctors *n* = 10, paramedics *n* = 11) giving a response rate of 47% overall.

All respondents reported that they were aware of the audible time calls at least some of the time, the majority (67%) describing this as “often”. The vast majority of clinicians (91%) agreed that the time calls had made them more aware of the passage of time on scene. Despite this, the majority felt this had not made a difference to scene times (62%) or changed their practice in any way (57%). Of the 9 who did feel their practice had changed 89% (*n* = 8) described this change with some positive level of acceptability.

Acceptability of the scene timer was viewed as either “somewhat” or “very acceptable” by 72% of all respondents, however 10% (*n* = 2) did find the timer “somewhat unacceptable”. 76% (*n* = 16) described having experienced some degree of timer-related distraction. Despite this only 2 respondents (10%) expressed the view that they would not want the use of the timer to continue.

## Discussion

Introduction of an audible scene timer into a HEMS service was associated with a statistically significant reduction in scene time for patients who require prehospital anaesthesia or have GCS < =8.

Experimental psychology has extensively studied the human perception of time and shown that perceived duration of time is affected by whether an individual is aware of the passage of time [18]. Research has shown that the less cognitive resource focussed on monitoring time, the shorter the period is perceived [18]. This same phenomenon has been demonstrated in clinical settings where clinicians have been found to significantly underestimate the amount of time elapsed whilst performing complex clinical interventions [13, 14].

We did not demonstrate a positive effect of prompting scene times for all undifferentiated single casualty trauma incidents. Although there was an overall trend towards lower scene times, specifically for blunt trauma, statistical significance was not reached. This may have various reasons. First, time awareness of clinicians is especially affected when their attention is focussed on (complex) clinical interventions [13, 14]. Therefore, it is likely that the largest effect of time prompting is to be expected in the subgroup of patients who undergo a prehospital intervention (such as prehospital anaesthesia). Second, besides time awareness, scene time is dependent on many other factors. Severity of injuries and resultant perceived urgency may play a role as well, as scene time has been shown to be longer for less injured patients [19, 20]. As we have not quantified perceived injury severity, this may have been a confounding factor. Finally, mode of transport to hospital was identified as a significant confounding factor, with longer scene times for patients transported by helicopter (often needing a secondary transport from scene to landing site). Although mode of transportation was entered as a co-factor in the pre- and post-intervention comparisons, we cannot completely exclude that this has affected our results, as mode of transport may also reflect differences in injury severity.

The tenant that time is a critical factor appears to have originated largely from expert opinion [21] and popularised by research findings from the Vietnam War where the 2% increase in survival compared to previous conflicts was attributed to reducing time to definitive care to 1 “golden” hour [22]. Although several studies have failed to substantiate the existence of a time and survival relationship for the undifferentiated and blunt civilian trauma cohorts [7, 23–25], this relationship has been established for patients with penetrating injuries, and patients with TBI [5–9].

In our study, we found a significant reduction is scene time (− 10.7%) for patients with a GCS < 8 and a similar reduction for patients requiring prehospital anaesthesia (− 17.6%). As GCS < 8 is a common finding in TBI and TBI is also a common indication for performing RSI, it is highly likely that a large proportion of the patients in both subgroups will also be TBI patients. As TBI patients as a subset have been found to benefit from reductions in prehospital time [8, 9], there is a clear potential for the introduction of the scene timer to improve clinical outcome for these patients.

Minimizing scene times is equally important for patients suffering from penetrating injuries [5, 6], as the number of interventions (and thereby the time on scene) in these patients is directly related to their mortality [6]. However, we could not demonstrate an effect of scene time prompting on scene times for these patients, as the number of patients with penetrating injury was small and our study was not powered to detect such difference. Furthermore, scene times for these patients are already relatively short (median 11 min), leaving less potential to for any intervention to improve these timings any further.

The use of the timer was found to be acceptable by the majority of clinicians who filled out the questionnaire and a majority felt this had made them more cognitively aware of the passage of time. However, most did not feel that the scene timer had affected their scene time or caused them to change practice. These perceptions are not supported by the actual scene time data describing a trend towards improving scene times. The number of clinicians reporting some degree of distraction is not surprising, as task fixation is common when completing complex tasks [13, 19]. It is likely that the distraction described is representative of the deliberate cognitive sharing created by the timer. This is supported by the respondents who describe an increased positive focus on time.

### Limitations

Our study has several limitations, most being inherent to the study design. First, in order to be able to compare the groups pre- and post-intervention, a large number of patients (multi casualty, entrapped, not transported) had to be excluded, which limits the generalizability of our study results. Second, analysis was on a per protocol basis, excluding 5 patients in whom the protocol was violated in the post-implementation period. Including these patients (in an intention to treat analysis) however, did not affect our results. Third, the proportion of patients treated by each individual clinician in each group was not evaluated. As individual clinicians vary significantly in terms of technical and experiential ability, this could have affected our findings, although the relatively small patient to clinician ratio makes such a performance bias unlikely.

Fourth, although subgroups were specified before data collection, no adjustment of sample size was made and therefore the risk of specifically type 2 error in subgroup analysis cannot be excluded. Further, we did not record efficiency or complication rate of procedures performed, nor did we have information on ISS scores or final outcome of our patients. As introducing a scene timer may put (additional) stress on clinicians on scene, these may have affected their ability to perform procedures as the delivery of prehospital anaesthesia. In addition, the response to the questionnaire was low (47%), thereby somewhat limiting the conclusions regarding feasibility and acceptability that can be drawn from it. Finally, we recognize that an audible timer may create distraction whilst at the same time it may have acted as a passive observer creating a perceived demand for performance thereby creating attention bias [26]. Whilst little is known of the mechanism and scale of such an “Hawthorne Effect” its existence has been conclusively established [27], and might have affected our findings.

## Conclusion

Audible scene timers may have the potential to reduce pre-hospital scene time for certain single casualty trauma patients treated by a HEMS team, particularly for those patients needing pre-hospital anaesthesia. Regular use of on-scene timers may improve outcomes by reducing time to definitive care for certain subgroups of trauma patients.

## Supplementary information

**Additional file 1: Supplementary file 1.** Study Questionaire.

## Data Availability

The datasets used and/or analysed during the current study are available from the corresponding author on reasonable request.

## Abbreviations

AAKSS: Air Ambulance Kent Surrey and Sussex
GCS: Glasgow Coma Scale
HEMS: Helicopter emergency Medical service
RSI: Rapid Sequence Induction
RTC: Road traffic collision
