# Supplementary figures and images for: The ticking clock: does actively making an enhanced care team aware of the passage of time improve pre-hospital scene time following traumatic incidents?
Source: Scand J Trauma Resusc Emerg Med. 2020 Apr 29;28:31. doi: 10.1186/s13049-020-00726-9 (PMC7189533; doi:10.1186/s13049-020-00726-9)

**Supplementary file 1.** Study Questionaire


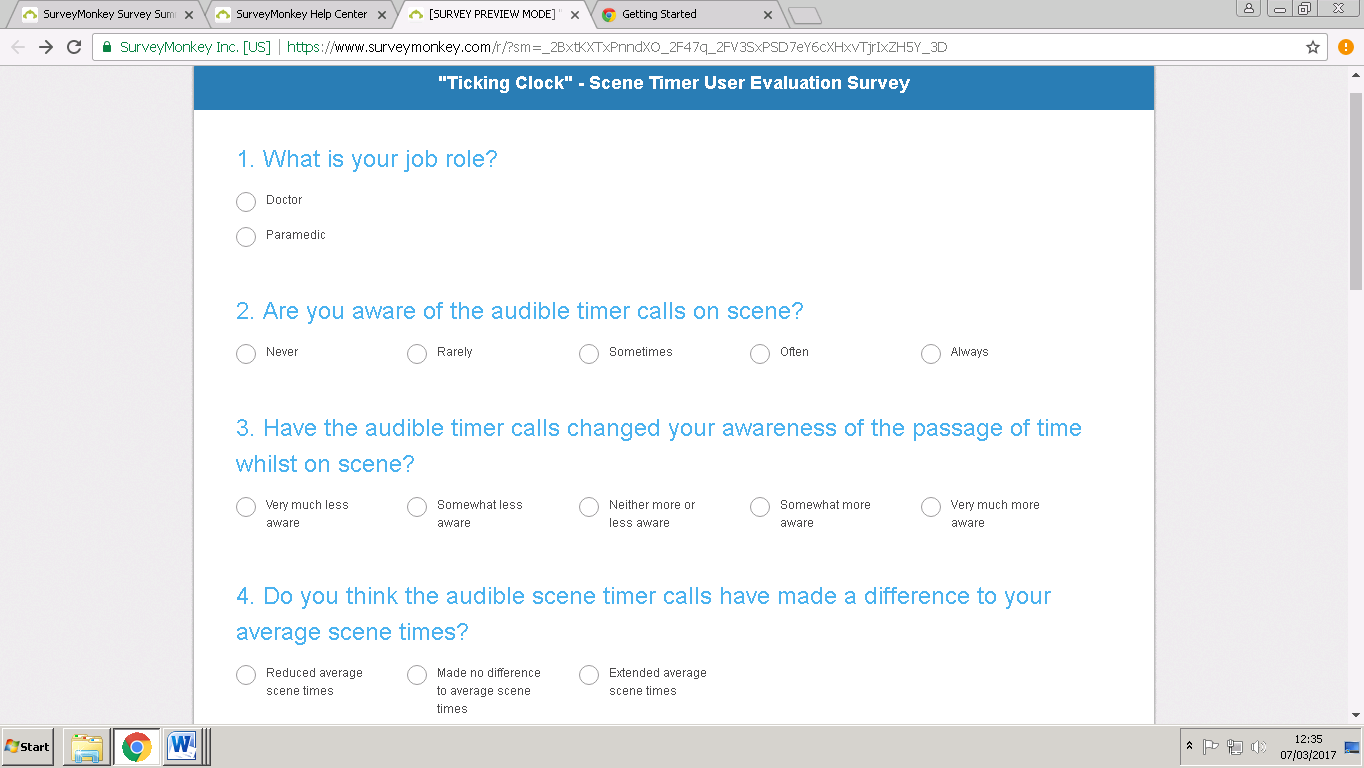


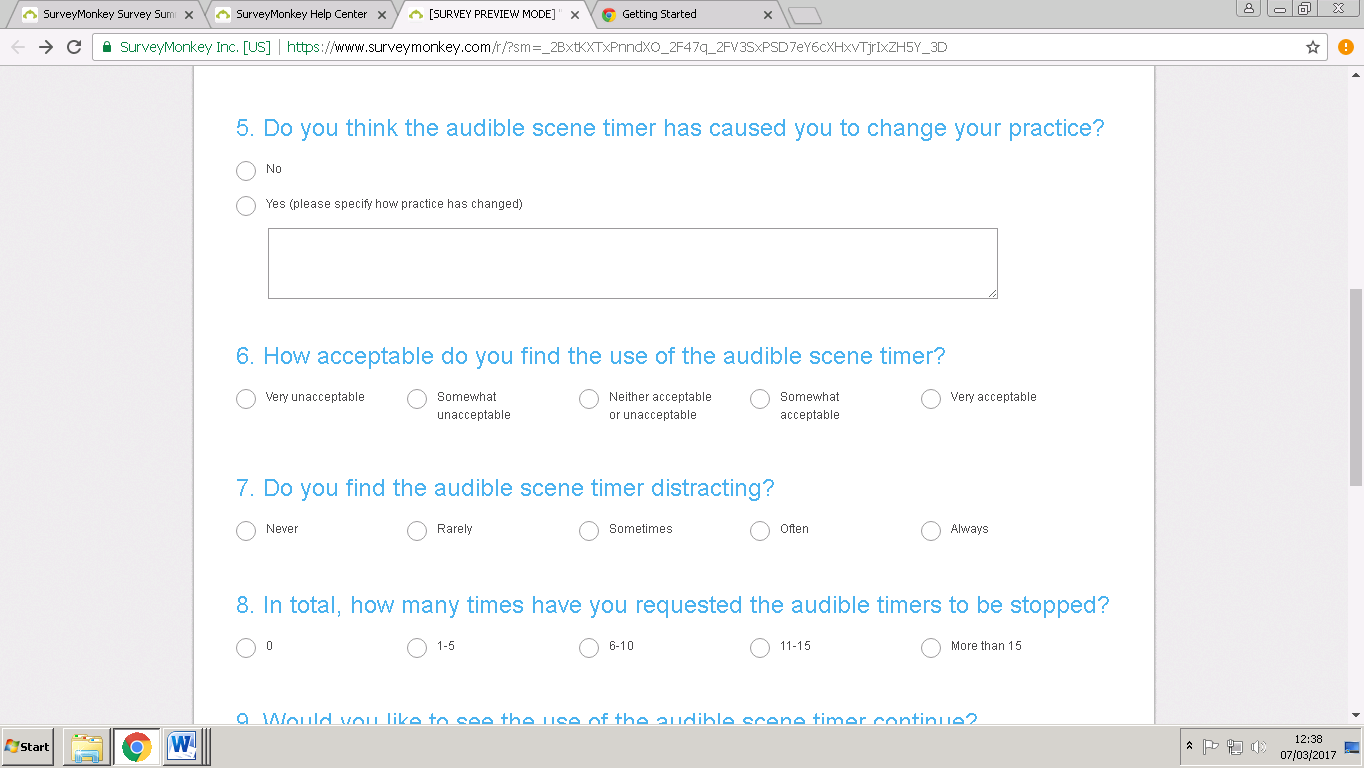


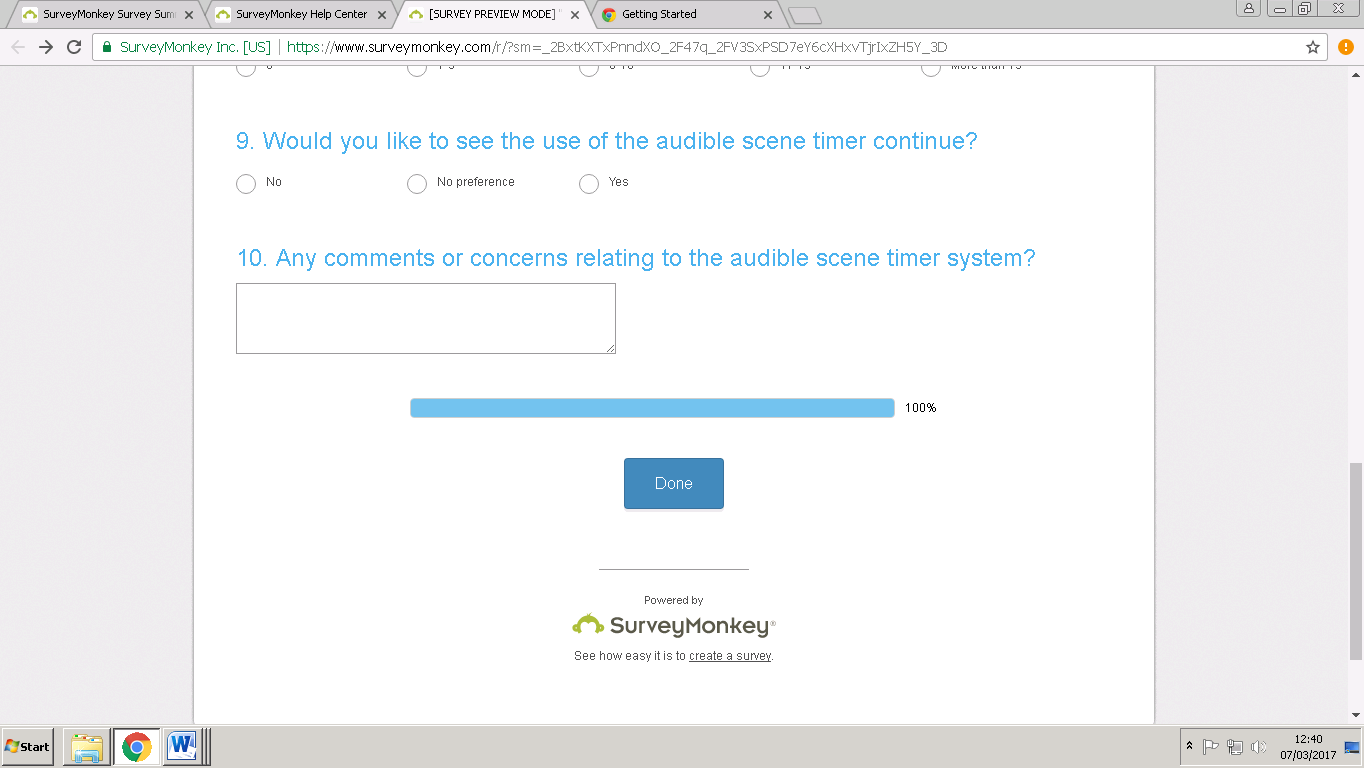

Supplement: Supplementary file 1 — Additional file 1: Supplementary file 1. Study Questionaire. [file 13049_2020_726_MOESM1_ESM.docx]
